# Antithrombin supplementation for anticoagulation during continuous hemofiltration in critically ill patients with septic shock: a case-control study

**DOI:** 10.1186/cc4853

**Published:** 2006-03-13

**Authors:** Damien du Cheyron, Bruno Bouchet, Cédric Bruel, Cédric Daubin, Michel Ramakers, Pierre Charbonneau

**Affiliations:** 1 Medical Intensive Care Unit, Caen University Hospital, Avenue côte de Nacre, 14033 Caen cedex, France; 2 Medical Intensive Care Unit, Bichat-Claude Bernard University Hospital, AP-HP, 46 rue Henri Huchard, 75018 Paris, France

## Abstract

**Introduction:**

Acquired antithrombin III (AT) deficiency may induce heparin resistance and premature membrane clotting during continuous renal replacement therapy (CRRT). The purpose of this study was to evaluate the effect of AT supplementation on filter lifespan in critically ill patients with septic shock requiring CRRT.

**Methods:**

We conducted a retrospective case-control analysis based on a 4-year observational study with prospectively collected data in two medical intensive care units in a university hospital. In all, 106 patients with septic shock underwent CRRT during the study period (55 during 2001 to 2002 and 51 during 2003 to 2004). Of these, 78 had acquired AT deficiency (plasma level below 70%) at onset of renal supportive therapy, 40 in the first 2-year period and 38 in the last 2-year period. In the latter intervention period, patients received AT supplementation (50 IU/kg) during CRRT each time that plasma AT activity, measured once daily, fell below 70%.

**Results:**

In a case-control analysis of the 78 patients with acquired AT deficiency, groups were similar for baseline characteristics, except in severity of illness as assessed by a higher Simplified Acute Physiology Score (SAPS) II after 2002. In comparison with controls, cases had a significantly greater AT level after AT supplementation, but not at baseline, and a smaller number of episodes of clots, without excess bleeding risk. The median hemofilter survival time was longer in the AT group than in the heparin group (44.5 versus 33.4 hours; *p *= 0.0045). The hemofiltration dose, assessed by the ratio of delivered to prescribed ultrafiltration, increased during intervention. AT supplementation was independently associated with a decrease in clotting rate, whereas femoral angioaccess and higher SAPS II were independent predictors of filter failure. However, mortality did not differ between periods, in the control period the observed mortality was significantly higher than predicted by the SAPS II score, unlike in the treatment period.

**Conclusion:**

In sepsis patients requiring CRRT and with acquired AT deficiency, anticoagulation with unfractionated heparin plus AT supplementation prevent premature filter clotting and may contribute to improving outcome, but the cost-effectiveness of AT remains to be determined.

## Introduction

The incidence of septic shock has increased drastically during past years. Septic shock patients have mortality rate of about 60% and an excess risk of death of about 25% when compared with non-septic patients [1]. Sepsis patients frequently develop endothelial damage and a hypercoagulable state related to the systemic inflammatory response syndrome [2]. In these severe situations, patients present acquired antithrombin III (AT) deficiency with plasma AT level lower than 80% either due to increased consumption related to disseminated intravascular coagulopathy (DIC) or induced by decreased liver synthesis, or increased vascular permeability and degradation by elastase [3]. A striking correlation between AT activity and survival in sepsis has been demonstrated [4-7]. Patients with multiple organ failure induced by septic shock need aggressive life support such as vasopressors, mechanical ventilation and/or renal supportive therapy. Continuous renal replacement therapy (CRRT) requires careful anticoagulation to prevent the blood from clotting while avoiding bleeding complications. Heparin treatment, especially in combination with extracorporeal circulation, may also lead to significant AT consumption [8], then to premature filter clotting despite adequate anticoagulation [9]. In 2000 Williams and colleagues [10] showed, in a randomized trial in patients requiring cardiopulmonary bypass, that heparin resistance was frequently associated with AT deficiency. Treating this deficiency with AT concentrate was more effective and faster for obtaining adequate anticoagulation than using additional heparin. Cardiopulmonary bypass is a traumatic procedure that is associated with platelet and coagulation defects, and with systemic inflammation, as described in septic shock. Thus we proposed that AT supplementation in the subset of septic shock patients undergoing CRRT might increase filter lifespan and improve the efficacy of this system of renal support.

## Materials and methods

### Setting and study cohort

**Figure 1:**
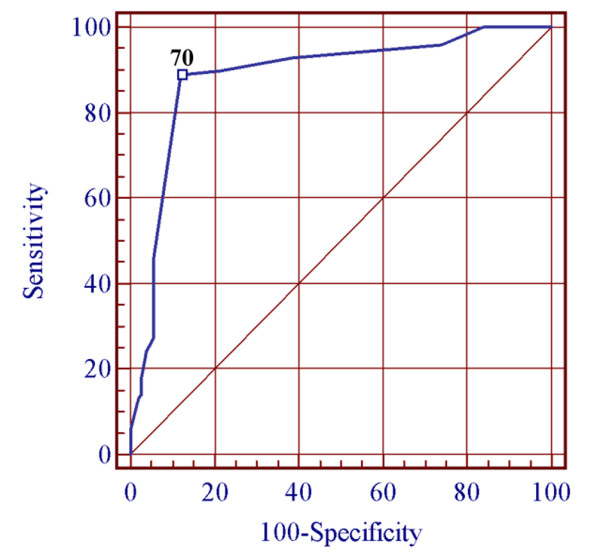
Receiver operating characteristic (ROC) curve for antithrombin in a group of septic shock patients (*n *= 55) who underwent continuous renal replacement therapy in the intensive care unit from January 2001 to December 2002. The ROC curve was generated by plotting sensitivity against (100 – specificity) for each value of AT. A threshold value of 70% with the highest sensitivity and specificity (88.9% and 87.9%, respectively) was set to predict filter clotting. The area under the curve is 0.886.

This retrospective study was conducted over a 4-year period (January 2001 to December 2004) in two 12-bed adult medical intensive care units (ICUs) in the University Hospital of Caen. A total of 106 patients with septic shock, as defined by the American College of Chest Physicians/Society of Critical Care Medicine [11], underwent CRRT for more than 24 hours during the study period. Demographic, clinical and laboratory data, including criteria for overt DIC according to the International Society of Thrombosis and Haemostasis DIC algorithm [12], as well as the Simplified Acute Physiology Score II (SAPS II) [13] and the Sequential Organ Failure Assessment (SOFA) score [14] to assess the severity of illness, were recorded prospectively in a computer database. From January 2001 to December 2002, 55 patients needed CRRT in the management of septic shock, with a crude filter clotting rate of 28.5%. Clotting was defined as a filter lifespan of less than 24 hours for those filters that were changed because of an increased drop in transmembrane or end-to-end pressure. In December 2002 we proposed that a decrease in filter lifespan may be associated with low plasma AT activity. We used a receiver operating characteristic (ROC) curve to determine the threshold value of AT concentration with the highest sensitivity and specificity to predict filter clotting. The area under the curve of the ROC curve constructed with plasma AT values of these 55 patients was 0.886, suggesting that AT level was a good predictor of filter clotting. From this ROC curve, the optimal cutoff that distinguished patients with a higher and lower risk of clotting was 70% (Figure 1). Indeed, the preliminary 40 patients with an AT activity of less than 70% had a greater frequency of filter clotting than the 15 patients with an AT activity of 70% or more (32% and 20%, respectively). Then sepsis patients requiring CRRT after December 2002 (the intervention period) were supplemented with AT if plasma activity level decreased below the cutoff value, following guidelines implemented in our ICU. Finally, only patients with AT activity of less than 70% during both periods were selected and compared in a case-control analysis. Ethical approval for this study was granted by the hospital ethical committee.

### CRRT directives

Department protocol for continuous veno-venous CRRT indications followed standard recommendations. CRRT (Prisma M100 preset AN69HF; Hospal, Lyon, France) was the technique of choice for hemodynamically unstable patients with suspected dialysis-induced hypotension; CRRT was then switched to intermittent hemodialysis as soon as possible. Angioaccess was achieved through the use of 12F double-lumen catheters inserted into the internal jugular or femoral veins. Blood flow was adjusted to between 150 and 200 ml/min, and ultrafiltrate at an outflow rate of 2 to 3 l/h was replaced with bicarbonate buffer solution. Hemofilters were primed with heparinized saline and changed every 72 hours. Systemic anticoagulation was performed with pre-filter unfractionated heparin (target for activated partial thromboplastin time (APTT) ratio 1.5 to 2.5 times normal; readjustment every 6 hours). Post-filter protamine (1 mg of protamine infused intravenously per 100 IU of heparin) was added for patients at high risk for bleeding, depending on the treating physician's judgment.

### Antithrombin supplementation

A blood sample was taken to measure plasma AT level at onset of CRRT in all patients. A control blood test was performed every day of treatment with continuous renal support. AT activity levels were determined with a chromogenic assay (bioMerieux antithrombin; bioMerieux, Marcy-l'Étoile, France; normal values 80 to 120%). The supplementation protocol sought to achieve a plasma AT level greater than 110 to 120%. Each time that AT activity dropped below 70% during the intervention period, 50 IU/kg AT (Aclotine; LFB, Les Ullis, France) were administered intravenously. The fixed daily 50 IU/kg dose regimen of AT supplementation was chosen because a 1.7% per IU/kg AT response and a mean half-life of 18.9 hours were expected in these patients, as reported [15].

### Evaluation of antithrombin efficacy

The primary endpoint was the rate of filter clotting during CRRT, defined as the number of clotting episodes divided by the number of treatment days. Secondary endpoints were the dose of hemofiltration (delivered to a prescribed ultrafiltration ratio, defined as the 24-hour true cumulative ultrafiltration volumes divided by the prescribed dose during the day), and the mortality.

### Statistical analysis

Values are expressed as means ± SD, median and range, or number and percentage as appropriate. Univariate analysis was performed with a χ^2 ^test and Fisher's exact test for categorical variables, and Student's *t *test or a Mann–Whitney test when appropriate for continuous variables. Survival curves for filters were prepared in accordance with the Kaplan–Meier method. The dependant variable (72 hours survival) was defined as success if the filter lifespan was greater than 72 hours. Backward deletion logistic regression analysis was performed on the population restricted to the 78 patients included in the case-control analysis to determine the set of independent predictors of filter clotting in patients with acquired AT deficiency. The dependant variable (filter clotting) was defined as success if circuit coagulation occurred more than once. We used *p *values of 0.1 to enter and remove variables from the model. The risk-adjusted mortality rate and 95% confidence intervals (95% CIs) were calculated. Analysis was performed with SAS 8.2 and MedCalc 7.4 statistical software. The two-tailed significance level was set at *p *< 0.05.

## Results

**Figure 2:**
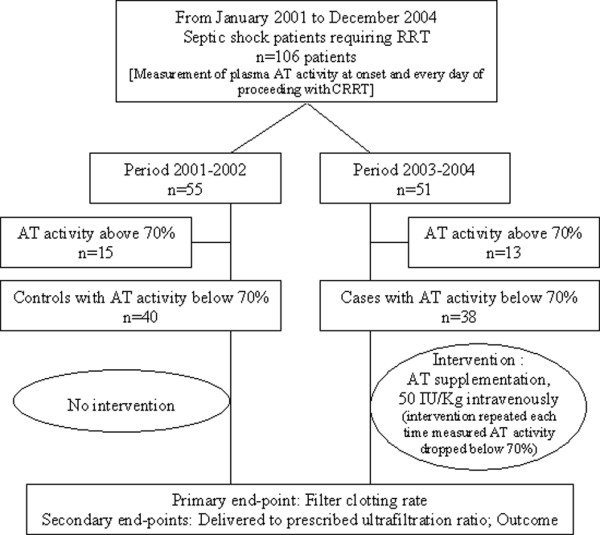
Study flow chart.

A total of 2,662 admissions of patients without septic shock were made in our ICUs over the study period. Ages and SAPS II scores were 54.8 ± 22.5 years and 38.6 ± 21.4, respectively, and the crude ICU mortality rate was 20.6%. During the same period, 230 admissions (7.9% of ICU admissions) concerned patients with septic shock (age 58.7 ± 20.2 years; SAPS II score 57.1 ± 22.0; ICU mortality 58.3%). Of these, 106 subjects (46%; age 59.6 ± 14.5 years; SAPS II score 58.2 ± 16.3) needed CRRT for a median duration of 4 days (range 1 to 9), with a crude filter clotting rate of 22% and a crude ICU mortality of 60% (Table 1). After exclusion of patients as described in Materials and methods (see Figure 2), 78 (74%) septic shock patients requiring CRRT were eligible for analysis.

In univariate analysis (Table 1), groups were similar for demographic data and co-morbidities. Modalities of CRRT, such as hemofiltration (36 patients) or hemodiafiltration (42 patients), did not differ between periods. Patients were more severely ill in the period 2003 to 2004, as assessed by higher SAPS II and SOFA scores at ICU admission. The number of overt DICs did not differ between the two periods, and AT activity was similar to baseline in both intervals; however, the mean AT activity reached a normal level within 24 hours after AT supplementation during the intervention period, and differed significantly from that of controls (95 ± 20% for cases, 55 ± 12% for controls; *p *= 0.001). During the intervention period, the median dose of AT received per patient was 50 IU/kg (range 50 to 150). Nineteen patients received a single dose, 13 received two AT doses, and 6 needed three AT infusions once daily, each dose being 50 IU/kg. AT supplementation was not associated with an increased risk of major bleeding events (*n *= 3 for cases; *n *= 2 for controls). The heparin dose required to achieve the targeted increase in APTT was lower during the intervention period (*p *= 0.0086). The frequency of post-filter protamine infusion was equal between groups (four controls versus five cases). Median lengths of CRRT and ultrafiltration rate were similar, but the filter clotting rate was significantly lower (*p *= 0.0018) and the ratio of delivered to prescribed ultrafiltration was significantly greater (*p *= 0.0032) in patients supplemented with AT.

**Figure 3:**
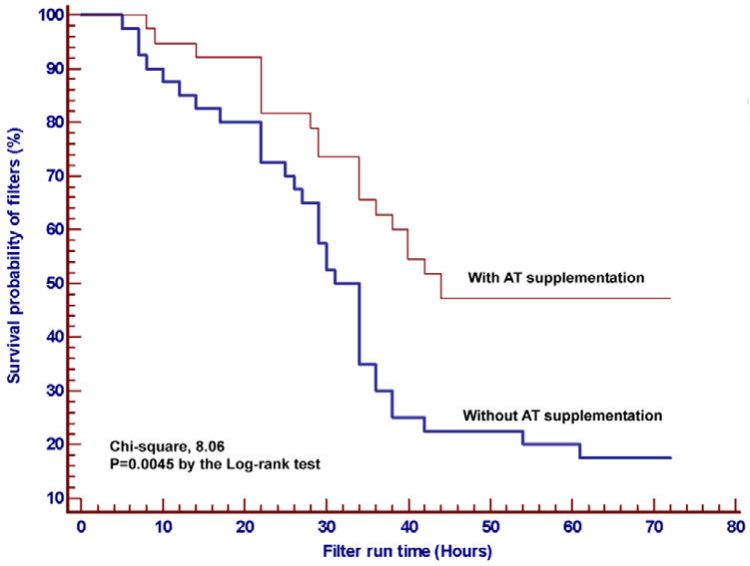
Survival curves of filters in patients with an AT of less than 70% according to AT supplementation (period 2) or not (period 1). The estimated hazard ratio was 2.15 (95% confidence interval 1.29 to 4.02).

As shown in Figure 3, the survival curve for filters in patients supplemented or not with AT concentrates differed significantly between periods. The median filter lifespan in patients who received AT was 44.5 hours (95% CI 34.5 to 48.0), which was significantly longer than the 32.5 hours (95% CI 26.5 to 36.0) in patients who received heparin alone (*p *= 0.0045 by the log-rank test).

By multivariable analysis adjusted for age, fibrinogen level, heparin dose, platelet count and the need for mechanical ventilation, AT supplementation was independently associated with a decrease in membrane failure, whereas higher SAPS II and femoral angioaccess were identified as independent predictors of clotting (Table 2).

Despite a greater severity of illness during the intervention period, the median lengths of ICU stay and the ICU mortalities did not differ significantly. When adjusted for the severity of illness, mortality for patients treated with AT did not differ significantly from that calculated in control patients (the risk-adjusted mortality rate was 1.1 for cases and 1.4 for controls, with overlapping confidence intervals). However, the observed hospital mortality rate was significantly higher than predicted mortality estimated by SAPS II during the period 2001 to 2002 (95% CI significantly different from 1), whereas the observed hospital mortality rate remained similar to the expected rate during the intervention period.

## Discussion

The recent conference on CRRT [16] failed to reach a consensus on the preferred anticoagulant for most CRRT patients. Systemic anticoagulation with unfractionated heparin remains the treatment of choice. Some authors have reported encouraging results with the concomitant administration of prostaglandin E_1 _[17], or fresh frozen plasma [18] and unfractionated heparin, to keep the circuit open. However, prostaglandin E_1 _requires adequate experience to avoid side effects, and transfusion with fresh frozen plasma presents the same risks as transfusion with red blood cells and is not routinely recommended during septic shock to correct laboratory clotting abnormalities in the absence of bleeding or planned invasive procedure [19]. More recently, a randomized trial has suggested the superiority of regional citrate anticoagulation over unfractionated heparin [20]. In this study, the median hemofilter survival time increased markedly from 38.3 hours in the heparin group to 124.5 hours in the citrate group. Decreasing AT levels were identified as independent predictors of hemofilter failure, and citrate anticoagulation was associated with a greater increase in AT over time after adjustment for temporal changes in illness severity. Finally, these results advocated the use of regional citrate as anticoagulation of choice in patients receiving CRRT, and highlighted the key role of AT levels to predict filter lifespan as described by others [8,9,21].

There are conflicting data on the use of AT in sepsis or extracorporeal circulation. On the one hand, the body of literature currently does not support the routine use of AT in sepsis patients despite encouraging results from experimental studies [22]. In 1998, a meta-analysis based on four double-blind placebo-controlled trials of patients with severe sepsis documented a non-significant 22% decrease in death rate in treated patients [23]. More recently, the KyberSept trial found no effect of AT on 28-day all-cause mortality in patients with severe sepsis or septic shock, despite a possible treatment benefit in the subgroup of patients not receiving concomitant heparin [24]. In this latter study, AT was associated with an increased risk of hemorrhage when it was administered with heparin. On the other hand, in heparin-resistant patients undergoing cardiac surgery with cardiopulmonary bypass, there are recent data suggesting that normalizing AT during extracorporeal circulation may modulate thrombin generation, decrease levels of fibrin monomer and D-dimer [25], restore heparin responsiveness, and then promote therapeutic anticoagulation [26,27].

Our observational study confirms the high incidence of septic shock patients with a lack of AT activity as well as the strong association between AT deficiency, heparin resistance and premature circuit coagulation. It also suggests that AT supplementation can effectively prevent the occurrence of clotting, at least in part by potentiation of the heparin effect on thrombin and factor Xa. This is substantiated by the decrease in heparin dose needed to achieve targeted APTT during the second two-year period. Finally, our results suggest that low AT levels have first to be supplemented to take advantage of a new membrane generation such as heparin-bonded filters.

Furthermore, as recently described by Lima and colleagues [28], our study confirms the difficulties in predicting outcome by using scoring systems at ICU admission in patients with acute renal failure. Mortality was underestimated by SAPS II in the control group of the present study and not in the treatment group. This could indicate changes in the management of treated patients that resulted in an improved outcome. The potential impact of AT supplementation must therefore be interpreted with caution. However, because patients were more severely ill during the intervention period, the benefit of AT on survival might have been underestimated, and AT might contribute to an improvement in outcome as assessed by the reduction of mortality adjusted for the severity of illness. In our study the ultrafiltration rate was about 35 ml/kg per hour and did not differ significantly between periods. However, the reduced downtime in treated patients contributed to an enhancement in the daily dose of hemofiltration in patients who received AT concentrates, as assessed by the ratio of delivered to prescribed ultrafiltration. Because a beneficial impact of higher dose of hemofiltration on survival of ICU patients with acute renal failure treated by continuous hemofiltration has been well demonstrated [29], this mechanism might be involved, at least in part, in improving the adjusted mortality risk in our study.

Another finding is the association between femoral angioaccess and circuit coagulation. The femoral vein is usually considered the primary choice in emergencies, whereas internal jugular access seems preferable in the absence of life-threatening thoracic conditions. Our results further support the placement of dialysis catheters in the internal jugular vein whenever possible, to decrease the rate of complications such as clotting and to improve the efficacy of hemofiltration. This should be confirmed by a well-designed randomized trial.

Some limitations of our study have to be addressed. Because the design was single-center and retrospective, with a comparison of unmatched small historical groups despite a prospective collection of data, we recognize that the conclusions drawn from the present study might not be generalizable. We simply reported the evolution of the incidence of filter failure between two periods during which only one element had changed: the supplementation of AT in patients with an endogenous AT activity of less than 70% during CRRT.

Finally, the cost of AT supplementation is high in our study. Despite a decrease in the mean number of filters used, AT (product and analysis) plus hemofilters in the second period cost about €730 per treatment day compared with €80 per treatment day for filters alone. However, the mean cost increase of €650 per treatment day can be counterbalanced by the time saved by nurses and intensivists, and by a potential beneficial effect on outcome. Thus, although AT supplementation represents a real cost to the hospital, further randomized studies are needed to evaluate its cost-effectiveness in septic shock patients treated with continuous hemofiltration.

## Conclusion

Our results suggest that AT administration in patients suffering from septic shock-induced multiple organ failure and requiring CRRT should be re-evaluated. As demonstrated in heparin-resistant patients necessitating cardiopulmonary bypass during cardiac surgery, AT supplementation may be used in the future as an alternative to regional citrate anticoagulation to improve the effect of heparin in patients undergoing CRRT during septic shock. Its safety, cost-effectiveness and impact on outcome remain to be determined by further double-blind, randomized, placebo-controlled multicenter trials.

## Key messages

• 	Acquired AT deficiency is frequently observed during septic shock.

• 	In septic shock patients undergoing CRRT, a low level of AT activity, with a threshold value of 70%, is associated with premature filter clotting.

• 	The body of literature does not recommend the routine use of AT concentrate to treat heparin resistance during CRRT.

• 	Our case-control observational study suggests that AT supplementation during CRRT restores heparin responsiveness, decreases the filter clotting rate and may improve outcome.

• 	Further double-blind, randomized, placebo-controlled multicenter trials are warranted to confirm these findings and determine the cost-effectiveness of AT supplementation.

## Abbreviations

APTT = activated partial thromboplastin time; AT = antithrombin III; CI = confidence interval; CRRT = continuous renal replacement therapy; DIC = disseminated intravascular coagulopathy; ICU = intensive care unit; ROC = receiver operating characteristic; SAPS II = Simplified Acute Physiology Score II; SOFA = Sequential Organ Failure Assessment.

## Competing interests

The authors declare that they have no competing interests.

## Authors' contributions

DdC conceived the original protocol, executed the study, analyzed data, and drafted the manuscript. BB, CB, CD, MR assisted in executing the study and drafting the final manuscript. PC participated in the coordination of the study. All authors read and approved the final manuscript.

## Figures and Tables

**Table 1:** Baseline characteristics for the overall population and for controls and cases with AT level below 70%

Characteristic	Overall population (*n *= 106)	Period 1 (2001–2002) (*n *= 40)	Period 2 (2003–2004) (*n *= 38)	*p*^d^
Age, years	59.6 ± 14.5	58.4 ± 14.5	60.5 ± 13.4	0.50
Male sex, *n*	70 (66)	27 (68)	27 (71)	0.81
Medical admission, *n*	85 (80)	33 (82)	30 (79)	0.78
Coexisting conditions, *n*				
Chronic liver disease	16 (15)	6 (15)	7 (18)	0.77
Immune deficiency	20 (19)	8 (20)	7 (18)	1.0
Chronic renal failure	9 (8)	2 (5)	4 (11)	0.42
Site of infection, *n*				0.93
Respiratory system	61 (58)	22 (55)	22 (58)	
Intra-abdominal	19 (18)	9 (22)	7 (18)	
Urinary system	15 (14)	4 (10)	3 (8)	
Other	11 (10)	5 (13)	6 (16)	
Microbial type, *n*				0.89
Gram-negative	22 (21)	9 (22)	9 (24)	
Gram-positive	32 (30)	10 (25)	12 (32)	
Other/mixed	15 (14)	3 (8)	3 (8)	
Unknown	37 (35)	18 (45)	14 (36)	
SAPS II	58.2 ± 16.3	55.2 ± 16.0	62.5 ± 16.1	0.047
SOFA score	8 (3–21)	8 (3–20)	10 (3–21)	0.012
Overt DIC, *n*	20 (19)	7 (18)	8 (21)	0.78
Need for mechanical ventilation, *n*	82 (77)	30 (75)	30 (79)	0.79
Length of vasoactive support, days	6 (1–15)	6 (1–15)	6.5 (1–14)	0.79
Time between ICU admission and onset of CRRT, days	1 (0–7)	1 (0–7)	1 (0–7)	0.86
Serum creatinine^a^, μmol/l	158 (82–480)	126 (94–380)	158 (82–480)	0.47
Blood urea nitrogen^a^, mmol/l	16.2 (5.5–57)	15.9 (8.8–50.6)	17.6 (6.2–57)	0.20
Fibrinogen^a^, g/l	6.2 ± 1.4	6.5 ± 1.3	6.5 ± 1.4	0.86
Platelets^a^, 10^3^/μl	114 (8–654)	107 (8–570)	92 (11–654)	0.80
Antithrombin activity level^a^, %	62.5 ± 19.1	53.5 ± 10.4	51.9 ± 11.2	0.53
Femoral angioaccess, *n*	66 (62)	25 (62)	21 (55)	0.65
APTT ratio^b^	2.2 ± 0.7	1.9 ± 0.5	2.0 ± 0.6	0.80
Heparin dose^b^, U/kg	771 ± 333	890 ± 389	683 ± 276	0.0086
Filter clotting rate, %	22	31.8 ± 25.0	16.5 ± 15.2	0.0018
Ultrafiltration rate, ml/kg per hour	34.1 ± 3.6	33.2 ± 3.4	34.4 ± 3.7	0.14
Ratio of delivered to prescribed ultrafiltration, %	83.1 ± 12.7	77.3 ± 12.3	86.1 ± 13.2	0.0032
Length of CRRT, days	4 (1–9)	4 (1–8)	4 (1–8)	0.85
Length of stay in ICU, days	10 (2–105)	8 (2–49)	11 (2–92)	0.22
Expected mortality, %	59.1 ± 24.1	54.0 ± 25.5	61.1 ± 23.8	0.21
ICU mortality, *n*	64 (60)	26 (65)	22 (58)	0.64
Hospital mortality, *n*	66 (62)	27 (68)	23 (60)	0.64
O/E ratio	1.1 [0.9–1.4]	1.4 [1.1–1.8]^c^	1.1 [0.9–1.4]	

^a^When initiating CRRT; ^b^means of APTT ratio and heparin dose during CRRT; ^c^95% confidence interval significantly different from 1; ^d^*p *value for univariate analysis between periods 1 and 2. Single numbers in parentheses are percentages; ranges are shown in parentheses; square brackets are used to indicate 95% confidence interval. APTT, activated partial thromboplastin time; CRRT, continuous renal replacement therapy; DIC, disseminated intravascular coagulopathy; O/E ratio, risk-adjusted mortality rate; SAPS II, Simplified Acute Physiology Score II; SOFA, Sequential Organ Failure Assessment.

**Table 2:** Set of independent factors associated with filter clotting determined by multivariable analysis

Factor	Odds ratio	95% CI	*p*
Antithrombin supplementation	0.21	0.06–0.74	0.0147
Femoral angioaccess	4.32	1.32–14.15	0.0156
SAPS II	1.06	1.01–1.12	0.0150

Backward deletion logistic regression analysis. Age, fibrinogen, heparin dose and platelets as continuous variables and the need for mechanical ventilation as a categorical variable were introduced into the model, then removed from the equation as described in Materials and methods. An odds ratio of less than 1 means a decreased risk of filter clotting. CI, confidence interval; SAPS II, Simplified Acute Physiology Score II.
